# mRNA vaccines encoding variant forms of Sm-TSP-2 confer protective immunity against Schistosoma mansoni

**DOI:** 10.21203/rs.3.rs-7237356/v1

**Published:** 2025-08-26

**Authors:** Athos Silva Oliveira, Sevan Alwan, Philip T. LoVerde, Ramiz Shaheed, Pranav Mandyam, Rakesh Adhikari, Peter Hotez, Maria Elena Bottazzi, Jeroen Pollet

**Affiliations:** National School of Tropical Medicine, Baylor College of Medicine; University of Texas Health Science Center; University of Texas Health Science Center; University of Texas Health Science Center; National School of Tropical Medicine, Baylor College of Medicine; National School of Tropical Medicine, Baylor College of Medicine; National School of Tropical Medicine, Baylor College of Medicine; National School of Tropical Medicine, Baylor College of Medicine; National School of Tropical Medicine, Baylor College of Medicine

## Abstract

Despite the global burden of helminth infections, no human vaccines have yet been licensed against these parasites. This study explored the development and evaluation of mRNA vaccine candidates targeting *Schistosoma mansoni* tetraspanin-2 (*Sm*-TSP-2), an antigen currently under evaluation as a protein vaccine. We designed constructs encoding either full-length *Sm*-TSP-2, or its large extracellular loop (EC2) domain in secretory, membrane-anchored, or cytosolic forms. In a murine challenge model, the secreted and membrane-anchored versions of *Sm*-TSP-2-EC2 induced the highest of antigen-specific antibody titers. These two construct designs, along with full-length *Sm*-TSP-2 mRNA, also significantly reduced adult worm and egg burden compared to controls. The membrane-anchored *Sm*-TSP-2-EC2 mRNA was the most effective, lowering worm and egg burdens by 66.7% and 66.9%, respectively. Protective responses by the mRNA vaccines were comparable to those induced by recombinant *Sm*-TSP-2-EC2 protein formulated with Alum. Histopathological analysis revealed smaller hepatic granulomas surrounding worm eggs, supporting the immunopathological benefit of vaccination. Using a systematic mRNA-based approach, we optimized the presentation of the *Sm*-TSP-2-EC2 and demonstrated that extracellular exposure of EC2 is essential for eliciting a protective immune response. These findings lay the groundwork for future development of multivalent mRNA vaccine strategies to achieve broader and more durable protection against schistosomes and other parasitic worms.

## Introduction

The development of vaccines against helminth parasites, such as hookworms and schistosomes, faces unique challenges due to significant biological and immunological barriers not commonly encountered with other pathogens [1–3]. Despite the complexity, significant progress has been made, with several anthelminthic vaccines advancing through clinical development for schistosomiasis, including a candidate targeting *Schistosoma mansoni* tetraspanin 2 (*Sm*-TSP-2) protein [4, 5].

Tetraspanins are a highly conserved family of transmembrane proteins with four-pass membrane domains and two extracellular loops: the small extracellular loop (EC1) and the large extracellular loop (EC2) [6]. *Sm*-TSP-2 plays an essential structural role in the development, maturation and maintenance of the worm tegument [7]. Due to the insoluble nature of full-length *Sm*-TSP-2, only its EC2 has been expressed as a recombinant protein [8–12]. Consequently, most studies employing recombinant *Sm*-TSP-2-EC2 refer to this domain simply as *Sm*-TSP-2. Recombinant *Sm*-TSP-2-EC2 has been used to generate antibodies that confirmed *Sm*-TSP-2 localization in the outer tegument of adult *S. mansoni*, and also has served as an antigen in both preclinical and clinical vaccine studies [10]. The *Sm*-TSP-2-EC2 structure has been resolved, enabling the identification of its interaction with other parasite proteins [13]. In mice, immunization with *Sm*-TSP-2-EC2 protein, formulated with Freund’s adjuvant, resulted in a 57% reduction of adult worm burden and a 64% reduction in liver egg burden [10]. In a phase 1, double blind clinical trial, conducted in a non-endemic area, an Alum-adjuvanted *Sm*-TSP-2-EC2 recombinant protein vaccine was safe and well tolerated [14]. Furthermore, in a randomized, controlled Phase 1b clinical trial involving healthy Brazilian adults living in an endemic region, the vaccine was safe, minimally reactogenic, and elicited significant IgG seroconversion [15].

While recombinant protein vaccines based on *Sm*-TSP-2-EC2 have shown immunogenicity and protection in schistosomiasis preclinical challenge models, further improvements in efficacy may still be achieved for optimal protective outcomes. Recent advancements in RNA technology have revolutionized the prevention and mitigation of infectious diseases by enabling the rapid development of effective, scalable, and customizable vaccines [16]. RNA vaccines facilitate the expression of full-length antigens and allow the incorporation of signal sequences that can modulate the antigen trafficking in recipient cells and tissues [17, 18]. While switching to an mRNA platform alone may not achieve sterilizing immunity, we hypothesized that *Sm*-TSP-2-based mRNA vaccines could induce protective efficacy comparable to or exceeding that of recombinant protein vaccines. The streamlined and antigen-independent manufacturing process of mRNA platforms makes them particularly suitable for multivalent vaccine strategies, either incorporating multiple schistosome antigens, or vaccines that can target multiple co-endemic parasites [19].

We recently developed mRNA vaccine candidates encoding hookworm antigen *Na*-GST-1, engineered for cytosolic accumulation, secretion, or plasma membrane (PM) anchoring [20]. Despite differences in protein expression among these constructs, antibody titers and T cell populations did not scale proportionally with protein levels, reinforcing that immune responses are more influenced by factors like antigen localization and processing.

In this study, we evaluated the efficacy of four mRNA vaccine candidates encoding either the full-length *Sm*-TSP-2 (native) or the *Sm*-TSP-2-EC2 domain targeted to different cellular compartments as cytosolic, secreted, or PM-anchored antigen. Mice were immunized with each mRNA formulation, and vaccine performance was evaluated by measuring *Sm*-TSP-2-EC2-specific IgG responses, as well as by assessing worm burden, egg burden, and hepatic granuloma size following challenge. A recombinant *Sm*-TSP-2-EC2 protein-based vaccine was included as a benchmark control.

## Results

### mRNAs enable the in-vitro expression of full-length Sm-TSP-2 and Sm-TSP-2-EC2 variants

**Figure 1:**
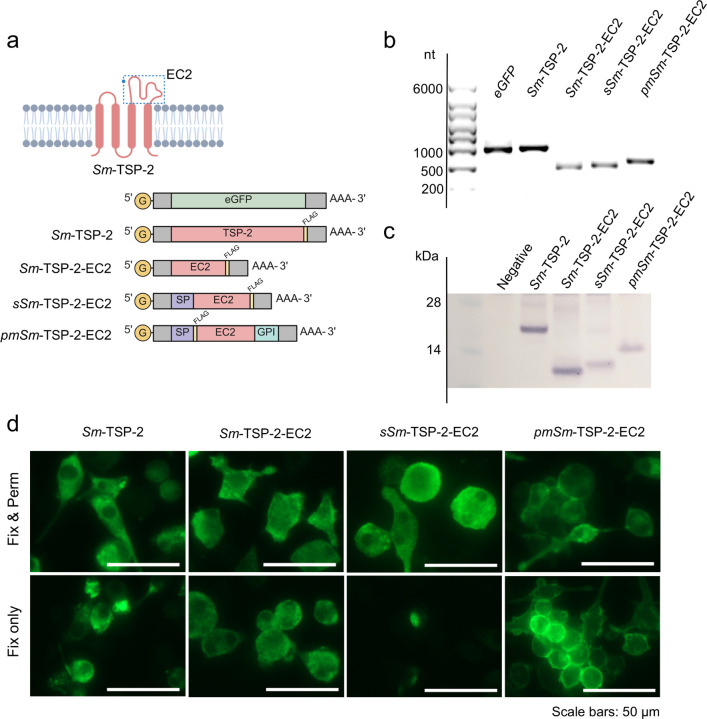
mRNA production and *in vitro* characterization. A) Schematic representation of *Sm*-TSP-2 showing its four-pass transmembrane domains and two extracellular loops, with EC2 highlighted by dashed lines. In the mRNA schemes, “G” represents CleanCap structure and grey regions indicate untranslated regions (UTRs). “SP” refers to signal peptide for protein secretion (*sSm*--TSP-2-EC2) or endoplasmic reticulum import (*pmSm*-TSP-2-EC2), while GPI indicates CD55 glycosylphosphatidylinositol signal sequence. Schematic created with Biorender.com. B) Integrity of synthesized mRNA assessed by agarose gel electrophoresis; 400 ng of each mRNA were loaded on a 1.5 % agarose gel. C) Confirmation of protein size by Western blot using anti-FLAG after transfection of mRNAs in DC2.4 cells. D) Subcellular localization of expressed proteins by immunocytochemistry in mRNA-transfected DC2.4 cells. In fixed and permeabilized cells, Alexa Fluor 488-conjugated mouse anti-FLAG can access all cellular compartments, including internal membrane-bound organelles. In contrast, in cells fixed without permeabilization (“Fix only”), the antibody is restricted to the cell surface and cytosol.

In addition to the Sm-TSP-2-EC2 mRNA, which encodes the same EC2 domain as the recombinant protein previously used in preclinical and clinical trials (referred to in this study as *rSm*-TSP-2-EC2 protein), two additional mRNAs were designed to investigate the impact of antigen localization: a secreted version (*sSm*-TSP-2-EC2), containing a signal peptide for secretion, and a PM-anchored version (*pmSm*-TSP-2-EC2), incorporating signal peptide for endoplasmic reticulum import and a CD55 glycosylphosphatidylinositol (GPI) anchor signal (Fig. 1A). A fourth construct encoding full-length *Sm*-TSP-2 mRNA was also designed. All mRNAs included a C-terminal FLAG, except *pmSm*-TSP-2-EC2, which contains an N-terminal FLAG to preserve proper detection by anti-FLAG. All mRNAs were successfully made in-house (Fig. 1B).

Following mRNA transfection in DC2.4 cells, Western blot (WB) analysis confirmed expression of proteins at their expected molecular sizes (Fig. 1C). Subcellular localization was observed by immunocytochemistry (Fig. 1D). Both *Sm*-TSP-2 and *Sm*-TSP-2-EC2 are localized predominantly to the cytosol, with *Sm*-TSP-2-EC2 also observed in the nucleus likely due to passive diffusion enabled by its small size (the nucleus is not seen in fixed-and-permeabilized cells). *sSm*-TSP-2-EC2 showed clear ER import as indicated by negligible intracellular staining in non-permeabilized cells. In contrast, *pmSm*-TSP-2-EC2 was clearly localized to the cell surface, consistent with successful GPI anchoring. Interestingly, the expression of *pmSm*-TSP-2-EC2 led to visible cell aggregation, potentially reflecting the role of *Sm*-TSP-2 as a tetraspanin scaffold protein. This suggests that the EC2 domain retains its native protein–protein interaction capabilities, even anchored by GPI.

### Sm-TSP-2 EC2 extracellular exposure by using signal sequences boosts IgG titers

**Figure 2:**
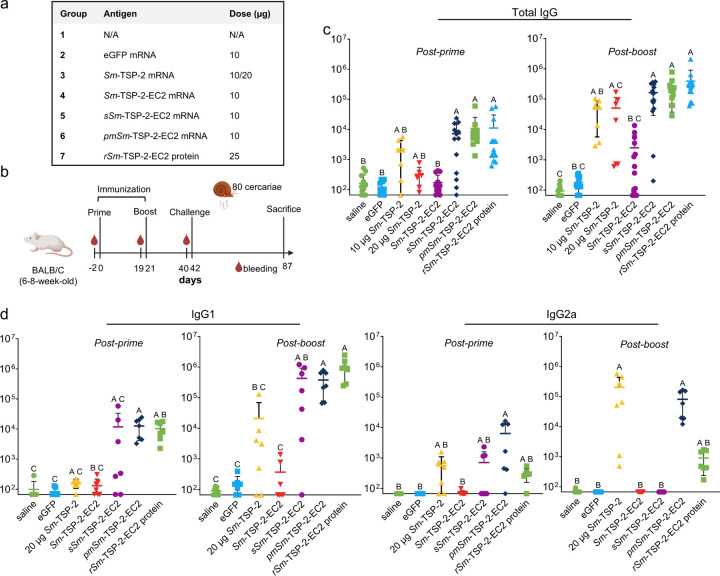
*Sm*-TSP-2-EC2-specific IgG responses following immunizations. A) Overview of the seven mouse groups, including the doses administered per immunization. B) Schematic timeline of immunization and challenge procedures (created with Biorender.com). C) *Sm*-TSP-2-EC2-specific IgG titers measured by ELISA. Results were pooled from two independent experiments, except for the *Sm*-TSP-2 group, which received a 20-μg equimolar dose (relative to *Sm*-TSP-2-EC2) in the second experiment. D) *Sm*-TSP-2-EC2-specific IgG1 and IgG2a titers measured by ELISA in samples from the second animal study. Each data point represents the mean of technical duplicates. Statistical analysis was conducted using the Kruskal-Wallis test followed by Dunn’s multiple comparisons test with correction for multiple testing. Groups that do not share a letter differ significantly (p < 0.05).

After *in vitro* validation, mRNAs were formulated into lipid nanoparticles (LNPs) as described in the Methods. To assess humoral immunogenicity and protective efficacy, 49 female mice were randomly assigned to seven groups (n = 7 per group) and immunized with one of the four *Sm*-TSP-2 mRNA vaccine candidates, or with *rSm*-TSP-2-EC2 protein formulated in Alum (Fig. 2A). Two control groups received either eGFP mRNA or saline. Blood samples were collected prior to the prime and boost immunizations, as well as before challenge with 80 S. *mansoni cercariae* (Fig. 2B). EC2-specific titers were measured in the mouse sera by ELISA using plates coated with *rSm*-TSP-2-EC2 protein. The experiment was independently repeated twice with one modification: in the first study, mice group 3 received 10 μg of *Sm*-TSP-2 mRNA, whereas in the second study, they received an equimolar dose of *Sm*-TSP-2 mRNA (20 μg) relative to the 10 μg *Sm*-TSP-2-EC2 mRNA dose. As no significant differences were observed between the two doses, data from both experiments were pooled for selected analyses presented throughout the manuscript.

*sSm*-TSP-2-EC2 and *pmSm*-TSP-2-EC2 mRNAs induced the highest antigen-specific IgG titers, comparable to those induced by the *rSm*-TSP-2-EC2 protein formulated with Alum (Fig. 2C). Although IgG titers were elevated in groups immunized with *Sm*-TSP-2 and *Sm*-TSP-2-EC2 mRNAs compared to the eGFP mRNA control, the differences did not reach statistical significance, indicating high variability or modest immunogenicity. These results collectively suggest that extracellular exposure, either by secretion or anchoring, is critical for robust humoral immunogenicity when *Sm*-TSP-2-EC2 is used as an antigen in mRNA vaccine formulations. It is important to highlight that ELISA plates were coated with *rSm*-TSP-2-EC2 protein, and therefore, the assay did not detect antibodies targeting epitopes outside the EC2 domains that may have been induced by *Sm*-TSP-2 mRNA.

Higher levels of human IgG1 and IgG3 against recombinant *Sm*-TSP-2-EC2 protein have been observed in the sera of putatively resistant individuals compared to chronically infected individuals in endemic regions of Brazil previously [10]. Given that IgG1 and IgG3 are generally associated with Th2 and Th1, respectively, the corresponding murine IgG1 and IgG2a subclasses were quantified in serum samples only from the second animal study (due to sample availability). The highest IgG1 titers were observed in mice immunized with *sSm*-TSP-2-EC2 and *pmSm*-TSP-2-EC2 mRNAs, and *rSm*-TSP-2-EC2 protein (Fig. 2D). Only *Sm*-TSP-2 and *pmSm*-TSP-2-EC2 mRNAs elicited statistically significant IgG2a titers compared to saline and eGFP mRNA. These findings support that intracellular antigen localization from *Sm*-TSP-2 mRNA favors a Th1 response, while GPI anchoring of *pmSm*-TSP-2-EC2 mRNA favors a balanced Th1/Th2 profile.

### pmSm-TSP-2-EC2 mRNA induces the greatest reduction in egg and worm burden after vaccination

**Figure 3:**
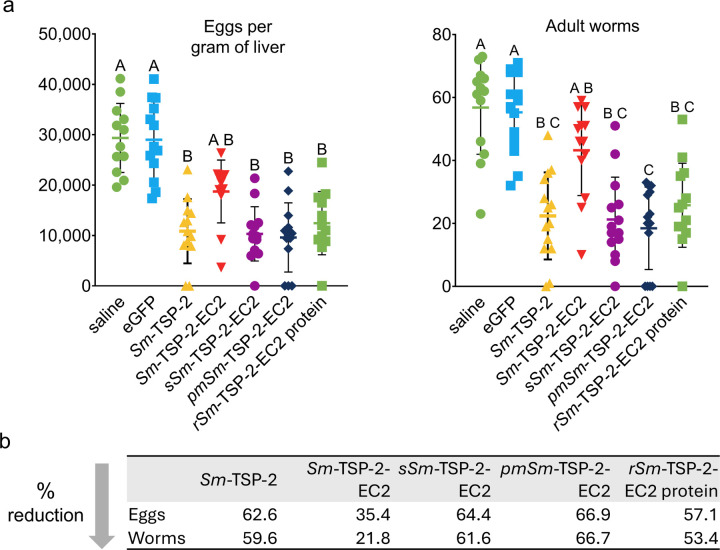
Reduction in egg and adult worm burdens following immunization. A) Parasite burden counts from two independent experiments. Statistical analysis was performed using the Kruskal-Wallis test followed by Dunn’s multiple comparisons test with correction for multiple testing. Groups that do not share a letter differ significantly (p < 0.05). B) Percent reductions in worm and egg counts were calculated relative to the eGFP mRNA group.

Three weeks after the boost immunization, each mouse was challenged with 80 *S. mansoni* cercariae and sacrificed 45 days later, corresponding to the time when parasites reach full maturity. Egg burden was determined by counting the number of eggs per gram of liver tissue, and adult worms were recovered through portal and mesenteric vein perfusion. All mRNA vaccine groups, except *Sm*-TSP-2-EC2 mRNA group, showed a significant reduction in egg burden compared to the saline and eGFP mRNA controls (Fig. 3A). A similar trend was observed for adult worm reduction, with *Sm*-TSP-2-EC2 being again the only group that did not significantly differ from the controls. Although not statistically different from the *sSm*-TSP-2-EC2 and *Sm*-TSP-2 mRNAs, or *rSm*-TSP-2-EC2 protein groups, *pmSm*-TSP-2-EC2 mRNA induced the greatest reduction in both egg and worm burdens based on percent reduction relative to the eGFP mRNA group (Fig. 3B). These findings are mostly paralleled with EC2-specific IgG titers, suggesting a correlation between humoral immune responses and parasite burden reduction. In contrast, the protective effect induced by *Sm*-TSP-2 mRNA appeared to involve immune responses targeting epitopes also outside the EC2 domain or not available when EC2 is expressed alone.

### Sm-TSP-2/EC2 mRNA vaccination may reduce granuloma size surrounding S. mansoni eggs

**Figure 4:**
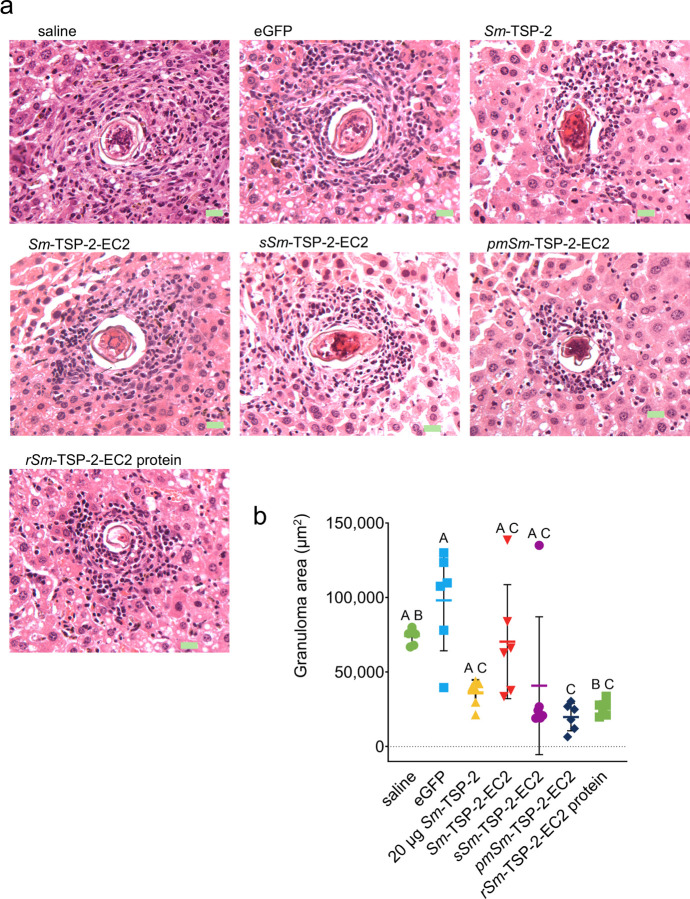
Morphological aspects and egg-induced hepatic granulomas from vaccinated and unvaccinated mice. **A)** Representative images of liver sections collected 45 days post-infection, stained with hematoxylin and eosin. Green rectangles indicate a scale of 20 μm. B) Data points indicate granuloma area (μm^2^) measured in liver sections. Statistical analysis was conducted using the Kruskal-Wallis test followed by Dunn’s multiple comparisons test with correction for multiple testing. Groups that do not share a letter differ significantly (p < 0.05).

Liver tissue from one representative mouse per group (second animal study) was examined for histopathological analysis. As expected, granulomas surrounding *S. mansoni* embryonated eggs were observed in all groups (Fig. 4A). However, a clear trend of reduced mean granuloma size was observed in mice immunized with *Sm*-TSP-2, *sSm*- TSP-2-EC2, *pmSm*-TSP-2-EC2 mRNAs, and *rSm*-TSP-2-EC2 protein (Fig. 4B). This reduction was statistically significant for *pmSm*-TSP-2-EC2 mRNA compared to both saline and eGFP mRNA groups. This decrease in granuloma size correlated with lower egg and worm burdens in vaccinated mice, suggesting that immunization with *Sm*-TSP-2/EC2 can reduce not only parasite load but also the pathological tissue responses associated with *S. mansoni* infection.

## Discussion

This study demonstrates that mRNA vaccines encoding full-length *Sm*-TSP-2 or *Sm*-TSP-2-EC2 can elicit antigen-specific antibody responses and reduce parasite burden in a murine model of *S. mansoni* infection. By engineering mRNAs to expose *Sm*-TSP-2-EC2 extracellularly, either through secretion or plasma-membrane-anchoring, we showed that both immunogenicity and protective efficacy are significantly enhanced compared to cytosolic EC2 expression. These responses were comparable to those induced by the benchmark recombinant *Sm*-TSP-2-EC2 protein formulated with Alum. These findings are particularly relevant for the inclusion of *Sm*-TSP-2-EC2 as a core component in future multivalent mRNA vaccine development strategies. Unlike protein subunit vaccines, which often require buffer compatibility and formulation optimization for co-formulating multiple antigens, mRNA vaccines offer greater flexibility by allowing co-formulation/delivery of multiple transcripts or the use of polycistronic constructs in a single injection.

To our knowledge, this study represents the first preclinical evaluation of full-length *Sm*-TSP-2 as a vaccine candidate. Previous recombinant protein-based vaccine studies have referred to the extracellular loop 2 domain (*Sm*-TSP-2-EC2) simply as *Sm*-TSP-2. Although (cytosolic) *Sm*-TSP-2 mRNA elicited stronger immunogenicity and protective efficacy than (cytosolic) *Sm*-TSP-2-EC2 mRNA, it did not outperform *sSm*-TSP-2-EC2 and *pmSm*-TSP-2-EC2 mRNAs. These results indicate that extracellular exposure of EC2 by the addition of signal sequences in mRNA vaccines greatly improved the humoral immunity and reduced parasite burden. Similar conclusions were drawn in other mRNA vaccine studies, where inclusion of signal peptides or GPI anchors improved antigen presentation and immunogenicity [18, 20, 21]. Our results also reinforce previous findings that EC2 harbors the dominant B-cell epitopes of *Sm*-TSP-2, and that expression of the *Sm*-TSP-2-EC2 is enough to induce protective immunity in mice [10, 22]. Further investigations are still needed to determine whether these vaccine candidates differ in their ability to induce durable T- and B-cell responses, which are critical for long-term protection.

In addition to *Sm*-p80, *Sm*14, *Sh*28GST, *Sm*-TSP-2 remains one of the few *Schistosoma* antigens that advanced through human clinical development [23–27]. Although some studies include the addition of TLR-4 agonists, most protein-in-adjuvant formulations are made with Alum, which predominantly induce Th2-skewed immune responses. Here, we demonstrated that *pmSm*-TSP-2-EC2 mRNA vaccine encoding membrane-tethered EC2 not only reproduces the immunogenicity of the subunit protein vaccine on Alum, but also promotes a more balanced Th1/Th2 profile, as evidenced by the presence of IgG2a, a Th1-associated subclass. Similar mixed subclass profiles have been reported in mice immunized with other GPI-anchored antigens [18, 20]. This Th1/Th2 also reflects patterns observed in humans naturally exposed to *S. mansoni* in Brazil, where individuals considered resistant exhibited higher levels of IgG1 and IgG3 against Sm-TSP-2-EC2 than those with chronic infections [10]. These findings suggest that especially *pmSm*-TSP-2-EC2 mRNA may more closely mimic protective immunity seen in naturally resistant individuals. Vaccination with either mRNA constructs or *rSm*-TSP-2-EC2 protein appeared to reduce hepatic granuloma size, with *pmSm*-TSP-2-EC2 mRNA inducing a statistically significant reduction. Similar findings have been reported in studies using *Sm*-TSP-2-EC2 (as a subunit protein or DNA vaccine) and *Sm*-p80, where reductions in worm and egg burdens were accompanied by attenuated granulomatous responses in the liver [28–30]. This effect may be due to decreased egg deposition, which limits antigen availability and subsequently reduces immune cell recruitment and cytokine production required for granuloma formation [31]. Alternatively, or in combination, *Sm*-TSP-2 vaccination may directly increase Th1 lymphocytes subsets, which release cytokines that help reduce granulomatous pathology by counteracting excessive Th2-mediated inflammation [32].

In conclusion, this work provides evidence that strategic design of mRNA constructs to control antigen localization can enhance both the magnitude and quality of the immune response against helminth infections. Given the continued need for effective schistosomiasis vaccines and the practical advantages of mRNA platforms, our results support further preclinical evaluation of these mRNA vaccine candidates. Future research should focus on long-term protection, cellular immunity, and dose optimization. Moreover, these vaccines should be evaluated in combination with other previously proven antigens, such as *Sm*14 and *Sm*-p80, as well as newly identified targets to explore potential synergistic effects and advance the development of a multivalent schistosome or multihelminth vaccines [33–36].

## Material and methods

### Ethic statement

All animal procedures were conducted in accordance with institutional and federal regulations, following review and approval by the UT Health San Antonio Institutional Animal Care and Use Committee under protocol #4061201100S7AR.

### RNA production

Human codon-optimized *Sm*-TSP-2 and EC2 (GenBank: FJ711440) were cloned into plasmid backbones containing T7 promoter, 5’ untranslated region (UTR), Kozak sequence, 3’ UTR, and poly(A) tail. Human IgG signal peptide was added upstream EC2 for antigen secretion. For PM-anchoring, EC2 was flanked by albumin signal peptide and a CD55 GPI attachment sequence. FLAG tag was added to the N-terminus (following the signal peptide) for PM-anchored EC2, and to the C-terminus of all other constructs. Plasmids were linearized and used as templates for in-vitro transcription following the protocol of CleanCap AG (Trilink Biotechnologies), and substituting rUTP for m1ΨTP. Template DNA was degraded with DNAse I-XT (New England Biolabs), and the synthesized mRNA was purified using silica-based spin columns. mRNA integrity was evaluated by incubating samples at 70 °C for 10 min and then ice for 5 min. Samples were loaded into 1.5% agarose gel.

### In vitro mRNA expression analysis

DC2.4 murine dendritic cells were cultured in RPMI 1640 + L-glutamine supplemented with 10% fetal bovine serum, 1 mM non-essential amino acids, 10 mM HEPES, 55 μM beta-mercaptoethanol, and antibiotics. Cells were maintained at 37°C in a humidified incubator with 5% CO_2_. For transfection, cells were seeded the day before and transfected with mRNA-Lipofectamine MessengerMAX complexes [Thermo Fisher Scientific (TFS)] according to the manufacturer’s instructions.

Complete protocols for immunocytochemistry (IC) and Western blot (WB) were published elsewhere [20]. Briefly, for immunocytochemistry, approximately 1.25 × 10^5^ DC2.4 cells were seeded per well in a 24-well plate and transfected with 250 μg mRNA. After 20 hours, cells were fixed with Cytofix Fixation Buffer or fixed and permeabilized with Cytofix/Cytoperm Buffer (BD, Franklin Lakes, NJ). After washing, cells were stained with Alexa Fluor 488-conjugated anti-FLAG monoclonal antibody (TFS, Cat. MA1–142-A488, 1:250) and imaged using an inverted fluorescence microscope.

For WB analysis, 2.5 × 10^5^ DC2.4 cells were seeded per well in a 12-well plate and transfected with 500 ng mRNA. Cells were lysed with RIPA buffer and protein concentration was quantified by BCA assay (TFS). In addition, samples were also resolved on SDS-PAGE gel, transferred, and probed with anti-FLAG M2 (MilliporeSigma, Cat. F3165, 1:1000) as primary antibody and alkaline phosphatase-conjugated goat anti-mouse (KPL, Cat. 5220 − 0357, 1:3000) as secondary antibody to confirm protein sizes.

### mRNA-LNP formulation and characterization

mRNAs were formulated into lipid nanoparticles (LNP) with Genvoy ILM lipid reagent (Precision Nanosystems) at a nitrogen-to-phosphate ratio of 4:1 on a NanoAssemblr Ignite^™^ instrument (Precision Nanosystems). The resulting mRNA/LNP complexes were concentrated using 30 kDa spin filter columns and sterilized by passage through 0.2 μm disk filters. Particle size and polydispersity were evaluated by dynamic light scattering using a Zetasizer Nano ZS90 (Malvern Panalytical), confirming an average LNP size of 90 nm and a polydispersity index below 15%. mRNA concentration was quantified using a RiboGreen RNA Assay kit (TFS). Detergent disruption with Triton X-100 verified that over 85% of the mRNA was successfully encapsulated in all formulations. Final vaccine formulations were diluted to 200 μg/ml mRNA in sterile 8% sucrose PBS.

### rSm-TSP-2-EC2 protein

*rSm*-TSP-2-EC2 protein was previously produced in *Pichia pastoris* as published elsewhere [8, 9]. For immunization, the protein was adjuvanted with Alhydrogel (Alum) (Croda) in a glucose-imidazole buffer (15% glucose, 2 mM phosphate, 10 mM imidazole, pH 7.4).

### Immunization, sampling, and challenge

Two independent replicate preclinical experiments were conducted. In each study, a total of 49 female BALB/c mice (Envigo/Charles River), aged 4–5 weeks, were randomly assigned into seven groups (n = 7 per group). Mice received a prime and a boost immunization, administered three weeks apart. mRNA vaccines were delivered via intramuscular injection at a volume of 50 μL per dose. The standard dosage was 10 μg per mouse, except for the *Sm*-TSP-2 mRNA, which was administered at 20 μg per dose in the second experiment. For protein immunization, recombinant Sm-TSP-2-EC2 (rSm-TSP-2-EC2) was administered at 25 μg per dose, adsorbed onto 160 μg of Alhydrogel.

Three weeks following the booster dose, mice were challenged percutaneously with 80 Schistosoma mansoni cercariae. Blood samples were collected via retro-orbital bleeding two days prior to each immunization and on the day of challenge. Post-infection, all mice were monitored daily for signs of pain or distress. At 45 days post-challenge, animals were euthanized using a sodium pentobarbital-containing solution administered at 200–250 mg/kg intraperitoneally, in accordance with the American Veterinary Medical Association (AVMA) guidelines. At the time of sacrifice, adult worms were recovered via perfusion of the portal and mesenteric veins. Livers were harvested for quantification of egg burden and for histopathological analysis.

### ELISA

Ninety-six-well flat bottom plates were coated overnight at 4°C with 100 μl of 0.25 μg/ml *rSm*-TSP-2-EC2 protein diluted in KPL coating solution (SeraCare Life Sciences). Plates were then blocked with 200 μl dilution buffer (0.1% BSA in PBST) for two hours at room temperature. After a single wash with PBST, wells were incubated in duplicate with 100 μl of serially diluted sera for 2 hours at room temperature. Mouse sera were serially diluted three-fold, ranging from 1:200 to 1:2,869,781,400 in dilution buffer. Naïve mouse serum was included in all plates, to establish background and cutoff values. Following incubation, plates were washed four times and incubated for 1 hour at room temperature with 100μl of HRP-conjugated secondary antibodies (goat anti-mouse IgG, IgG1 or IgG2a; Lifespan Bioscience) in dilution buffer. After five washes, 100 μl TMB substrate was added to each well and developed for 15 min. Reactions were stopped with 100 μl 1M HCl, and absorbance was measured at 450 nm using a BioTek Epoch 2 spectrophotometer (Agilent). Duplicate readings were averaged, and titers were calculated using a four-parameter logistic regression curve. The titer cutoff was defined as the mean absorbance of naïve serum controls plus three times the standard deviation. Data was plotted and statistical analyses were performed using GraphPad Prism software.

### Adult worm and egg counts

Harvested worms were cultured in 2ml DMEM supplemented with 10% FBS and antibiotic-antimycotic solution. Worms were manually sorted and counted under a dissecting stereomicroscope as previously described [37]. To estimate the egg burden, livers were removed, weighed, and 1 mg of each was digested in 10 ml of 5% KOH overnight at 37°C. The number of eggs per gram of tissue was determined by light microscopy at 40x magnification as previously described [38]. Data was plotted and statistical analyses were performed using GraphPad Prism software.

### Histopathology analysis

After perfusion, livers (one representative per group) were harvested, fixed in 70% ethanol, and processed for hematoxylin and eosin staining. Liver histopathology was assessed based on granuloma structure and size. To quantify average granuloma size, six well-defined granulomas per liver were selected, excluding fused granulomas (containing more than one egg) and those with unclearly visible or destroyed eggs. Tissue sections were examined under a Zeiss Axio Imager Z1 microscope, and measurements were performed by outlining granuloma boundaries at 4x magnification using ZEN software measurement tool for automatic area calculation. Data was plotted and statistical analyses were performed using GraphPad Prism software.

## Data Availability

All data generated or analyzed during this study are included in this published article.
